# Perceived Impact of Electronic Medical Records in Physician Office Practices: A Review of Survey-Based Research

**DOI:** 10.2196/ijmr.2113

**Published:** 2012-07-28

**Authors:** Jesdeep Bassi, Francis Lau, Mary Lesperance

**Affiliations:** 1 School of Health Information ScienceUniversity of VictoriaVictoria, BCCanada; 2 Department of Mathematics and StatisticsUniversity of VictoriaVictoria, BCCanada

**Keywords:** Health care surveys, evaluation studies, ambulatory care information systems

## Abstract

**Background:**

Physician office practices are increasingly adopting electronic medical records (EMRs). Therefore, the impact of such systems needs to be evaluated to ensure they are helping practices to realize expected benefits. In addition to experimental and observational studies examining objective impacts, the user’s subjective view needs to be understood, since ultimate acceptance and use of the system depends on them. Surveys are commonly used to elicit these views.

**Objective:**

To determine which areas of EMR implementation in office practices have been addressed in survey-based research studies, to compare the perceived impacts between users and nonusers for the most-addressed areas, and to contribute to the knowledge regarding survey-based research for assessing the impact of health information systems (HIS).

**Methods:**

We searched databases and systematic review citations for papers published between 2000 and 2012 (May) that evaluated the perceived impact of using an EMR system in an office-based practice, were based on original data, had providers as the primary end user, and reported outcome measures related to the system’s positive or negative impact. We identified all the reported metrics related to EMR use and mapped them to the Clinical Adoption Framework to analyze the gap. We then subjected the impact-specific areas with the most reported results to a meta-analysis, which examined overall positive and negative perceived impacts for users and nonusers.

**Results:**

We selected 19 papers for the review. We found that most impact-specific areas corresponded to the micro level of the framework and that appropriateness or effectiveness and efficiency were well addressed through surveys. However, other areas such as access, which includes patient and caregiver participation and their ability to access services, had very few metrics. We selected 7 impact-specific areas for meta-analysis: security and privacy; quality of patient care or clinical outcomes; patient–physician relationship and communication; communication with other providers; accessibility of records and information; business or practice efficiency; and costs or savings. All the results for accessibility of records and information and for communication with providers indicated a positive view. The area with the most mixed results was security and privacy.

**Conclusions:**

Users sometimes were likelier than nonusers to have a positive view of the selected areas. However, when looking at the two groups separately, we often found more positive views for most of the examined areas regardless of use status. Despite limitations of a small number of papers and their heterogeneity, the results of this review are promising in terms of finding positive perceptions of EMR adoption for users and nonusers. In addition, we identified issues related to survey-based research for HIS evaluation, particularly regarding constructs for evaluation and quality of study design and reporting.

## Introduction

The importance of office-based electronic medical records (EMRs) and related systems is being recognized internationally. For example, Canada Health Infoway [1] has an investment program to support the adoption and use of EMRs to help clinicians achieve increased clinical value. In its 2001 report entitled “Crossing the Quality Chasm,” the Institute of Medicine discussed using information technology as one aspect of improving the health care delivery system in the United States [2]. Until now, adoption of EMRs in the ambulatory setting has been relatively slow [3,4]. According to the 2007 National Physician Survey, only 12.3% of Canadian family practitioners and general practitioners used EMRs exclusively, and 19.4% used a combination of EMRs and paper-based charts [5]. These figures rose to 21.5% and 27.5%, respectively, in the 2010 National Physician Survey [6], which indicates that adoption is on the rise. Ford et al [3] constructed a model using historical data to estimate that 86.6% of physicians in small practices will be using the systems in 2024 in the United States.

Given what appears to be a slow but increasing trend of EMR adoption, the next area that needs attention is the impact of such systems to both ensure that they are adopted and that they are helping practices to realize the expected benefits. Talmon et al [7] stated that “given the essential role of information technology (IT) systems on the delivery of modern health care, and the dependence of health professionals and organizations on them, it is imperative that they are thoroughly assessed through robust evaluations as with any other form of health process or technology” (p. 23). Evidence of positive effects of EMRs is still limited [8]. In terms of guiding impact assessment, the recently developed Clinical Adoption Framework by Lau et al [9] provides a comprehensive set of categories that address many areas for overall health information systems (HIS) adoption.

Taking a closer look at impact, we see that it can be evaluated objectively (for example, by using proxy measures such as reduction in medication errors), but there is also a subjective component for individuals involved with EMR adoption. EMRs are expected to have positive impacts in many areas, but do providers believe this? Ultimate acceptance and use of the system is up to the provider, so there is a need to understand their point of view. Based on the trends presented above, there are two general views to consider: nonuser/preimplementation and user/postimplementation. Those who already have an EMR are able to share their perceived experienced impact of use, whereas those who don’t will have perceived expected impacts (ie, perceived benefits or concerns) that may hinder or drive adoption. One way to collect the views of users and nonusers is through the use of surveys. Surveys are commonly used in information systems evaluation [10-12].

In this review, we specifically address survey-based research studies. Surveys, or questionnaires, refer to the actual instruments used to gather data within a survey-based research study [10,13], which is an overall study design methodology. A survey instrument can be used as one data collection method within another methodology, but here we focused on studies where a survey was the primary means of gathering data. This review offers three contributions to researchers and practices planning to use this approach in future evaluation studies. First, it determined which areas of EMR impact have been evaluated using surveys and which areas have not. This provides an indication of what future survey research could address or where there are prior results available for comparison. Second, it describes a detailed approach, addressing the recognized challenge of reconciling results across heterogeneous studies [14,15], to synthesize the results and present summaries of views for the most prominent impact areas. Third, it contributes to the knowledge regarding survey-based research in the context of HIS evaluation and highlights quality issues to help inform the design of future studies. Therefore, the three key questions for this review are as follows. (1) What areas of EMR impact have been addressed most in survey-based papers (and subsequently, which areas have received little attention)? (2) For those areas that have been addressed most, what have the subjective views been so far regarding the perceived impact of EMRs among users and nonusers in some key areas? (3) How have survey-based studies been designed and used so far, and what are some common quality issues that should be considered?

## Methods

### Paper Selection

We briefly summarize the search strategy here, with selection flow details available in Multimedia Appendix 1. This review initially began as part of a larger systematic review on the impact of EMRs on physician office practices. In consultation with a librarian, we constructed queries for two online databases: Medline and CINAHL. As well, citations from systematic reviews on HIS were considered. Preliminary screening was carried out by one reviewer, followed by a full-text review done by two teams of two reviewers. Final selection decisions were made by consensus. Included papers had to evaluate the perceived impact of using an EMR system and its clinical functionality in an office-based practice, be based on original data, have providers as the primary end user, and report outcome measures that related to the positive or negative impact of the system. Since not all papers used the term *electronic medical record *and some used other terminology for such systems, we made decisions based on descriptions provided in papers and whether they discussed clinical functionality associated with an EMR. In this review we followed the definition provided by the Canadian Medical Protective Association [16] that an EMR generally refers to an electronic version of the paper record and is specifically used in ambulatory physician practices with added functionality to support clinical care. Please note that we use the term *electronic medical record *in this review for consistency when discussing papers regardless of the term used in the original paper. Papers were excluded if they were studies of outpatient EMRs integrated with inpatient information systems or conducted at hospital-based outpatient clinics, had patient respondents, reported on the same data as another paper, or had inadequate reported data on outcome measures, or if it was not possible to distinguish relevant results. At this stage, the set of selected papers included analytic, descriptive, and survey-based studies on the impact of EMRs on office-based practices. A separate review was published on the set of analytic and descriptive papers [17], and the remaining survey-based studies were included in this review. For this review, we extended our search to include survey-based studies published between 2000 and 2012 (May).

### Quality Assessment

In terms of design quality, we considered methods and reporting quality as well as the constructs for evaluation. For study methods design quality, we used the set of 9 survey methodological attributes developed by Grover et al [18], where those with an asterisk can be assessed further for strength of study design: (1) report the approach used to randomize or select samples*, (2) report a profile of the sample frame, (3) report characteristics of respondents, (4) use a combination of personal, telephone, and mail data collection, (5) append the whole or part of the questionnaire, (6) adopt a validated instrument or perform a validity or reliability analysis*, (7) perform an instrument pretest, (8) report on response rate*, and (9) perform a statistical test to justify the loss of data from nonrespondents*. Although these attributes were originally designed for management information systems survey research, we believe they are applicable to the HIS context as well. To score papers, each attribute is given a score of 1 or 0 depending on whether it is present in the paper or not, respectively. The final score for each paper is then determined by adding all attribute scores. Like the creators of survey methodological attributes, we assumed that unreported attributes were not performed and assigned a score of 0. A limitation noted for the survey methodological attributes is that the dichotomous measures don’t capture richness of some variables. We modified the scoring in our review to allow attributes to be given a score of 0.5 if they were somewhat present or weak. Scoring was done independently by two reviewers, and final scores for each paper were determined through consensus. All papers were included in the review for the insights they offered, but it is important for the reader to be aware of quality shortcomings. Ju et al [19] used survey methodological attributes to highlight the quality of research and considered a journal article to be adequate if the total survey methodological attributes score was greater than 4.5 out of a possible 9. We used the same interpretation. The intent here is to demonstrate how different papers addressed the 9 attributes and to highlight areas for improvement across the HIS evaluation field.

### Data Extraction

**Figure 1:**
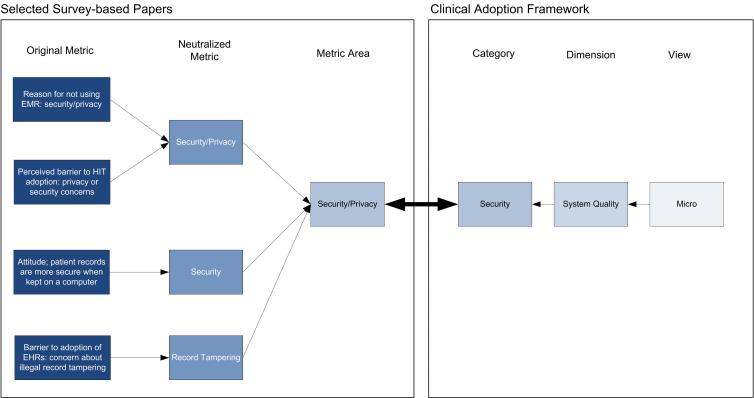
Example mapping of metrics to the Clinical Adoption Framework. EHR = electronic health record, EMR = electronic medical record, HIT = health information technology.

For the first step in the data extraction process, we identified all survey items and questions from each paper, which we termed *metrics*. For example, privacy and confidentiality concerns as a potential barrier to EMR adoption were one metric. The next step was to organize all these metrics in a way that would facilitate meta-analysis. The challenge in combining data from several papers that address a common area is reconciling the constructs used for evaluation within each paper. According to Ju et al [19], “a critical process in the maturing of any discipline is the development of proper constructs and instruments to collect adequate and accurate data about phenomena of interest.” To determine these constructs for synthesis, we mapped all extracted metrics to categories of the Clinical Adoption Framework (see Multimedia Appendix 2). The Clinical Adoption Framework and its predecessor, the Benefits Evaluation Framework, are based on DeLone and McLean’s Information System Success Model [9]. The Clinical Adoption Framework groups evaluation categories into dimensions, which are then organized according to three views: meso, macro, and micro. The meso view is concerned with people, the organization, and implementation as a whole. The macro view considers environmental factors that have a direct influence on categories in the meso view such as standards, funding, and societal trends. Finally, the micro view is focused on the user level and net benefits in specific areas where the system is expected to have an impact. In 2003, Van Der Meijden et al [20] reviewed evaluations of patient care information systems and categorized them using DeLone and McLean’s framework. They identified attributes from each paper and placed them into the framework categories. For our review, a metric area is equivalent to an attribute and is defined as the general aspect being evaluated, such as security. After extracting the reported metrics, we classified them into general metric areas and mapped them to Clinical Adoption Framework categories to allow for comparison and synthesis. Figure 1 shows how this was done. This mapping identified the main constructs for evaluation addressed in the papers. Finally, the last step was to split the corresponding results according to nonusers and users where possible. The aim was to extract only the data related to EMRs based on clinical functionality. Some papers included results on general information technology use such as Internet or email. We did not extract data pertaining to these except in cases where general functionality was embedded into clinical functionality.

### Meta-Analysis

The goal of the meta-analysis was to identify the most commonly addressed areas and combine the reported results for these areas to determine users’ and nonusers’ overall views toward EMRs. We determined that the raw data presented in some papers needed to be transformed to make them comparable. The first step was to consider whether the metric was posed as negative or positive so that the reported results could be interpreted as either negative or positive.

The surveys collected two types of data: dichotomous (ie, proportions or percentages for agreement with statements) and categorical (eg, Likert-type scale scores), and they were not reported in the same manner in all papers. For the dichotomous data, if the result was not already expressed as a proportion, we calculated a proportion estimate based on the sample size reported in the paper. As well, some papers divided results into further groupings within the nonuser and user categories, so we pooled these where possible using 95% confidence intervals to confirm an overlap for pooling. We created a series of 2 × 2 tables to organize the reported results for each metric with respect to positive and negative views for users and nonusers. Using the tables, we calculated the estimated odds of a perceived positive view for users and nonusers and then, where possible, an estimated odds ratio for a positive view for users to nonusers. For the categorical data, we redefined the scales used in the papers where needed to make mean values comparable. Most papers used a 5-point scale, but it was sometimes reversed or used different values. We transformed each scale so that it ranged from 1 (strongly negative) to 5 (strongly positive). Mean scores were recorded for nonusers and users where possible. The resulting odds calculations and mean scores were interpreted and compared with reported findings in the papers to determine overall perceived views for each selected area. Positive views leaned toward more perceived benefits of the potential use of systems, whereas negative views represented more perceived concerns or barriers that could possibly hinder use.

## Results

### General Characteristics of Selected Papers

We selected 19 survey-based papers for inclusion in the review [21-39], presenting both user and nonuser views (see Table 1). Several papers presented results for both categories [21,22,25-27,29,33-35,37,38], 1 looked only at preimplementation [36], and a few were only postimplementation views [23,24,30-32,39]. The implementation state of respondents’ EMR systems was not clear in 1 paper [28].

**Table 1:** Papers reporting results for the two categories of use status.

Author (year)	Preimplementation/ nonusers	Postimplementation/ users	Not specified
Chiang et al (2008) [21]	X	X	
DesRoches et al (2008) [22]	X	X	
Devine et al (2010) [23]		X	
El-Kareh et al (2009) [24]		X	
Gans et al (2005) [25]	X	X	
Johnston et al (2002) [26]	X	X	
Kemper et al (2006) [27]	X	X	
Leung et al (2003) [28]			X
Loomis et al (2002) [29]	X	X	
MacGregor et al (2006) [30]		X	
Mackenzie (2006) [31]		X	
Magnus et al (2002) [32]		X	
Menachemi et al (2007) [33]	X	X	
Russell and Spooner (2004) [34]	X	X	
Simon et al (2007) [35]	X	X	
Simon et al (2008) [36]	X		
Simon et al (2008) [37]	X	X	
Singh et al (2012) [38]	X	X	
Terry (2005) [39]		X	

Table 2 presents the general characteristics reported in the papers. In terms of respondents, the majority of surveys were administered to the physicians themselves [22-24,26,29,30-34], including specialists [33,39] such as pediatricians [27,34] and ophthalmologists [21]. In some cases, the respondents were the entire practice [25,38], nurses [23,31], or administrative office staff [37].

Many papers aimed to determine the perceived impact of adoption [21,22,24,25,27,35,36,39]. Others were specifically concerned with assessing perceived barriers to adoption [22,25,27,29,32-35,37,38], benefits [21,22,25,27,28,30,38], or overall attitudes toward adoption or use [22-24,26,27,29,31,32,34,36]. A few looked at physician satisfaction in general [21,22,39] and 2 focused on specific functionality [31,32]. (Note that these categories are not mutually exclusive.) Of the selected papers, 4 considered information and communication technology in general [26,30,36] but described features of EMRs, so we included them as well.

**Table 2:** General paper characteristics.

Author (year)	Country	Survey/study objective(s)	Respondents	Clinical context (ie, setting)	Survey method	Total sample	Response rate
Chiang et al (2008) [21]	United States	Assess the state of EHR^a ^use by ophthalmologists, including adoption rate and user satisfaction	Ophthalmologists	Medical practices	Web-based survey (with 2 email reminders) and telephone survey	3796	15.6% (592)
DesRoches et al (2008) [22]	United States	Assess (1) physicians’ adoption of outpatient EHRs, (2) satisfaction with such systems, (3) perceived effect of the systems on the quality of care, (4) perceived barriers to adoption	Physicians	Physicians providing direct ambulatory patient care	Mailed questionnaire (2 reminders by mail and phone); cash incentive	5000 (4484 eligible)	62% (2758)
Devine et al (2010) [23]	United States	Identify prescriber and staff (end user) characteristics that would predict attitudes and behaviors toward e-prescribing adoption in the context of a preexisting EHR	Prescribers (physicians, physician assistants, nurse practitioners) and staff (nurses and medical assistants)	3 primary care sites	Administered at the sites with 2 reminders sent via email	Total of 188 opportunities	Overall: 62% (117); prescribers: 82%; staff: 50%
El-Kareh et al (2009) [24]	United States	Measure changes in primary care clinician attitudes toward an EMR^b ^during the first year following implementation	Physicians, nurse practitioners, physician assistants	Ambulatory health centers	Mailed questionnaire at 1, 3, 6, and 12 months postimplementation (2 mailings and reminder emails)	73 physicians; 10 nurse practitioners; 3 physician assistants	Month 1: 92% (79); month 2: 95% (81); month 3: 90% (76); month 12: 82% (69)^c^
Gans et al (2005) [25]	United States	Assess the rate and process of adoption of information technology and EHRs by medical group practices	Group practices	Group practices with 3 or more physicians practicing together with a common billing and medical record system	Web-based and mailed survey; a subset of nonresponders were surveyed by phone	17,195	21.1% (3628)
Johnston et al (2002) [26]	China	Identify prevailing attitudes among physicians to use of computers in the clinical setting and specifically those attitudes that may be associated with the adoption of computers in practice	Physicians	Individual practices	Mailed questionnaire	4850	18.5% (897)
Kemper et al (2006) [27]	United States	(1) Measure penetration and functionality of EMRs in primary care pediatric practice, (2) identify plans for adoption of EMRs, (3) understand common barriers to adoption, (4) evaluate attitudes toward EMRs among those with and without one	Pediatricians	Office-based practice	Separate mailed questionnaires to those with and without an EMR (3 mailings); cash incentive	1000 (901 eligible)	58% (526)
Leung et al (2003) [28]	China	Understand the contributory barriers and potential incentives associated with information technology implementation	Physicians	General physician population (individual and corporate settings)	Mailed survey (3 mailings and maximum of 7 phone calls)	949	77% (731)
Loomis et al (2002) [29]	United States	Investigate possible differences in attitudes and beliefs about EMRs between EMR users (early market) and nonusers (mainstream market)	Family physicians	Active members in the Indiana Academy of Family Physicians	Mailed survey (2 mailings)	1398	51.7% (618 usable)
MacGregor et al (2006) [30]	Australia	(1) Examine perception of benefits derived from information technology adoption, (2) determine whether practice size, number of patients treated, gender of practitioner, or level of computer skills of the practitioner are associated with the perception of benefits	General practitioners	General practice	Mailed questionnaire	690	17.7% (122)
Mackenzie (2006) [31]	New Zealand	Nurses’ and doctors’ perceptions of the introduction and subsequent use of the Medtech 32 clinical module	Nurses, doctors	Family planning clinics	Paper questionnaire	132	57% (47 nurses and 28 doctors)
Magnus et al (2002) [32]	England	(1) Assess general practitioners’ views on the relevance of information provided by computerized drug interaction alert systems, (2) determine the proportion of general practitioners who admit to frequently overriding alerts without properly checking them, (3) explore factors that might be associated with a tendency to override alerts	General practitioners	Primary care trust areas	Mailed questionnaire (2 mailings)	336	70% (236)
Menachemi et al (2007) [33]	United States	1. Examine rural–urban differences in the use of various information technology applications by physicians in the ambulatory setting	Physicians (family medicine, internal medicine, pediatrics, obstetrics and gynecology)	Ambulatory settings	Mailed questionnaire (2 mailings)	14,921	28.2% (4203)
Russell and Spooner (2004) [34]	United States	(1) Determine the use of EMRs in area practices, (2) identify physicians’ attitudes adopting EMRs, particularly differences in attitudes between users and nonusers and between internal medicine and pediatric clinicians	Physicians (internal medicine and pediatrics)	Medical outpatient practices of internal medicine and pediatrics	Faxed and mailed survey (3 faxes and mailing); cash incentive	51 internal medicine, 24 pediatrics	Internal medicine: 51% (26); pediatrics: 63% (15)
Simon et al (2007) [35]	United States	(1) Determine the degree to which physicians used the various functions available in their EHR systems, (2) identify factors that correlate with use	Physicians	Office-based practice	Mailed survey (3 mailings with phone calls in between); cash incentive	1921 (1884 eligible)	71.4% (1345)
Simon et al (2008) [36]	United States	(1) Assess the degree to which the MAeHC^d ^practices are representative of physician’ practices statewide, (2) assess practice characteristics related to EHR adoption, prevailing office culture related to quality and safety, attitudes toward HIT^e^, and perceptions of medical practice	Physicians	Physician office practices	Mailed survey with multiple reminders	MAeHC: 464; statewide: 1884	MAeHC: 77% (355); statewide: 71.4% (1345)^f^
Simon et al (2008) [37]	United States	(1) Determine the state of EHR adoption and the degree to which doctors with EHRs are using the functionalities of those systems, (2) assess whether practices that had not yet adopted EHRs planned to adopt such systems and when, and what barriers impeded their progress	Office practice managers	Active medical and surgical practices (hospital and non-hospital based)	Mailed questionnaire (2 mailings and 2–6 phone calls)	1829	46% (847)
Singh et al (2012) [38]	United States	(1) Examine HIT and EMR adoption and use among primary care offices across the rural–urban spectrum, (2) assess perceived benefits and perceived barriers and facilitators to adoption	Offices (targeted office medical directors or owners)	Primary care offices	Mailed survey (reminder and second mailing); cash incentive	4669	21.4% (1001)
Terry (2005) [39]	United States	Determine EHR penetration, satisfaction, and use	Medical doctors and doctors of osteopathic medicine (including family practitioners, general practitioners, internists, obstetricians and gynecologists)	Office-based practice	Mailed survey	10,000	Not reported

^a ^Electronic health record (term used in the paper).

^b ^Electronic medical record.

^c ^Only included month 12 data in analysis.

^d ^Massachusetts eHealth Collaborative.

^e ^Health information technology.

^f ^Only included Massachusetts eHealth Collaborative data in analysis, as statewide data are reported in Simon et al [35].

### Quality

Using the survey methodological attributes, we deemed more than half of the papers (12) to be of adequate quality (see Table 3). The items with the highest average scores were reporting a profile of the sample frame, with a response rate, and a profile of respondents. For sample frame we looked for inclusion and exclusion criteria that identified the target sample. In many cases the sample frame was all clinicians belonging to a membership or organization. Most papers reported a response rate and respondent characteristics in a table. A few papers provided demographic information at the practice level, rather than the individual respondent level, so we scored these as 0.5 for this item. The item that scored most poorly across all papers was analyzing the reliability or validity of item measurement or adopting a validated instrument. For this item we specifically looked for an indication of an analysis done to confirm the validity or reliability of instrument questions. Many papers reported developing instruments in consultation with experts, basing the questionnaire on previous work, or having an expert panel review it. However, only 1 paper [29] specifically reported having a test-retest reliability rate for each item. The other item that generally scored low was for the use of a combination of personal, telephone, or mail data collection. In most cases, survey data were collected solely through a mailed questionnaire. A few papers reported an opportunity for respondents to complete the survey by Web or telephone as well. We scored these as 1.

**Table 3:** Quality assessment using the survey methodological attributes.

Author (year)	Criteria items^a^									Total score^b^
	1	2	3	4	5	6	7	8	9	
Leung et al (2003) [28]	0.5	1	1	1	1	0.5	1	1	1	8
Chiang et al (2008) [29]	0.5	1	1	1	1	0.5	1	1	0	7
Singh et al (2012) [38]	1	1	0.75	0	1	0	1	1	1	6.75
DesRoches et al (2008) [22]	0.5	1	1	0	1	0	1	1	1	6.5
Gans et al (2005) [25]	1	1	0.5	1	1	0	0	1	1	6.5
Magnus et al (2002) [32]	1	1	1	0	0.5	0	1	1	1	6.5
Devine et al (2010) [23]	0.5	1	1	0.25	1	1	0.5	1	0	6.25
Loomis et al (2002) [29]	1	1	1	0	0	1	1	1	0	6
Menachemi et al (2007) [33]	0.5	1	1	0	0	0.5	0.5	1	1	5.5
Simon et al (2008) [37]	1	1	0	1	0	0	0	1	1	5
MacGregor et al (2006) [30]	1	0.5	1	0	1	0	0	1	0	4.5
Simon et al (2007) [35]	0.5	1	1	1	0	0	0	1	0	4.5
El-Kareh et al (2009) [24]	1	1	1	0	0	0	0	1	0	4
Kemper et al (2006) [27]	0.5	1	0.5	0	0	0	1	1	0	4
Russell and Spooner (2004) [34]	1	0.5	0.5	0	1	0	0	1	0	4
Simon et al (2008) [36]	1	1	0.5	0	0	0	0	1	0	3.5
Johnston et al (2002) [26]	0.5	1	1	0	0	0	0	1	0	3.5
Mackenzie (2006) [31]	0.5	1	1	0	0	0	0	0.5	0	3
Terry (2005) [39]	0	0	0	0	0	0	0	0.5	0	0.5

^a ^1 = sample selection approach, 2 = profile of sample frame, 3 = respondent characteristics, 4 = data collection methods, 5 = sample of questionnaire, 6 = validation of instrument, 7 = instrument pretest, 8 = response rate, 9 = test for nonrespondent.

^b ^Out of a possible maximum score of 9.

### Reported Metric Areas

During the data extraction phase of the review, we pulled reported metrics from the papers and grouped them into more general metric areas under the categories of the Clinical Adoption Framework. Only those metrics related to EMRs were extracted, which excluded general information technology. However, metrics related to use of other clinical information technology were extracted into the category of information and infrastructure. For example, Gans et al [25] asked respondents about use of other computer-based information systems, and Simon et al [37] reported whether having computerized claims or billing systems, computerized scheduling systems, or computerized prescribing systems was associated with adoption. Table 4 provides an overview of the mapping of metric areas addressed in the papers to categories of the Clinical Adoption Framework. The most-addressed categories were personal characteristics, structure and processes, stage, appropriateness and effectiveness, efficiency, and functionality. These appeared to have received the most attention in the surveys reported. Sometimes a paper had multiple metrics for a category. For a more detailed analysis, we split the metric areas into three groups: background, impact-specific, and other. Each group is described in the section below.

Several Clinical Adoption Framework categories did not have any metric areas identified: individual and groups, roles and responsibilities, added values, legislative acts, political trends, economic trends, use behavior pattern, intention to use, and participant and caregiver participation.

#### Background Areas

Most background areas corresponded to categories under the meso level, since surveys often had items pertaining to the background of respondents and practices such as practice size (number of staff), system use status, gender, future intention to use a system, and specialty. These areas and their metrics were often used not only to describe the sample, but also to determine whether there were any correlations within the reported findings. For instance, several papers found that system adoption and use was greater in larger practices [22,25,27,33,35]. We chose use status as the main categorical variable for our meta-analysis to help split results into nonuser/preimplementation and user/postimplementation categories.

#### Other Areas

We also extracted all other areas addressed that related to EMR adoption. These were not determined to be specifically impact related but were other associated aspects of EMR implementation that have been addressed through surveys. Table 4 shows that most tended to correspond to categories under the macro level. Expense of implementation and functionality, either available or desired according to respondents, came up frequently. We included the use of features as a separate area, as availability of a feature doesn’t necessarily correspond to use. A few papers [22,35,37] made this distinction.

#### Impact-Specific Areas

The third group of addressed areas were the ones of interest for this review. These areas specifically addressed the perceived potential or actual impacts of implementing and using an EMR. As shown in Table 4, most impact areas corresponded to categories at the micro level as expected, specifically under the net benefits dimension. No impact-related metrics mapped to the macro level, but a few did map to categories in the meso level. It is important to note there would not necessarily be impact-specific areas for every category of the Clinical Adoption Framework because it encompasses all aspects of system adoption.

**Table 4:** Mapping of metric areas to clinical adoption framework.

Level	Dimension	Category	Metric area	Type^a^	Papers (reference number)	Total number of metrics
Meso	People	Individuals and groups	(Determined by type of respondent survey is administered to)		All	0
		Personal characteristics	Age	B	23, 26, 28, 29, 31, 32, 33, 36, 39	9
			Gender	B	22, 23, 24, 26, 28, 29, 30, 32, 33, 35, 36	11
			Race and ethnic background	B	22	1
			Income	B	28	1
			Active in general practice and status	B	35	2
			Graduation year and years of practice	B	22, 24, 26, 34, 35, 36	6
			Specialty	B	22, 23, 26, 28, 33, 34, 35, 36, 37, 38, 39	11
			Computer skills and literacy	B	23, 26, 30, 31, 34, 36	9
			First to have new tests or treatments (general practice)	O	36	1
		Personal expectations	Comparison between paper based and electronic	I	27	1
			Feelings toward practice in general	O	35, 36	8
			Protecting physicians from personal liability for record tampering by external parties	I	22	1
		Roles and responsibilities				0
	Organization	Strategy	Actively improving quality (general practice)	O	36	1
			Local physician champion	O	38	1
			Physician recruitment	I	25	1
		Culture	Bad previous experience with an electronic record system	O	27	1
			Attitude toward the electronic record system	I	22, 24, 25, 26, 27, 29	4
			Physician and staff resistance	O	36, 37	9
			Isolation from colleagues (general practice)	O	35, 36	2
			Innovative staff (general practice)	O	36	2
		Information and infrastructure	Ability to interface and integrate with existing practice systems	O	21, 25, 27, 39	6
			Technical limits	O	36	1
			Use of other clinical information technology	O	25, 37, 38	4
		Structure and processes	Practice size (number of staff)	B	21, 22, 25, 26, 27, 28, 29, 30, 33, 34, 35, 36, 37, 38, 39	18
			Practice size (number of patients)	B	24, 29, 30, 35, 36	5
			Practice size (number of offices)	B	38	1
			Time spent caring for patients (hours)	B	24, 26, 28	3
			Practice type (eg, group)	B	26, 28, 33	3
			Remuneration patterns	B	26, 28	2
			Practice setting (eg, hospital or medical center)	B	22, 37	2
			Type of office	B	23, 38	4
			Patient population	B	38	2
			Practice location	B	22, 29, 33, 36, 37, 38	7
			Communication with general practice business suppliers	O	30	1
		Return on value	Business expansion	I	30	1
			Expense of implementation	O	21, 22, 25, 26, 27, 28, 29, 33, 36, 37, 38	13
			Maintenance costs	O	21, 27, 26, 29, 33, 36	7
			Expected return on investment	I	22, 25, 27, 33, 34, 38, 39	7
	Implementation	Stage	Use status	B	21, 22, 25, 26, 27, 29, 32, 33, 34, 35, 36, 37, 38, 39	16
			Future intention to use	B	21, 22, 23, 27, 33, 34, 37, 38, 39	12
		Project	System development or selection	O	21, 22, 25, 27	5
			Time costs associated with computerization	I	21, 25, 26, 28, 33, 36	7
			Loss of productivity during transition	I	22, 33, 36, 38	5
			Entering historical data	O	25	1
		HIS^b^–practice fit	Staff requirements for implementation and maintenance	O	26, 27	2
			Meeting needs and requirements	O	22, 25, 27, 33, 37	5
			Capital available for practice expansion	O	36	1
Macro	Health care standards	HIS standards	Standardized medical terminology	O	21	1
			Transience of vendors	O	27	1
			Uniform data standards within the industry	O	25, 33, 36	3
		Performance standards	Evaluation of changes to improve quality (general practice)	O	36	1
			Quality problems (general practice)	O	36	1
			Procedures and systems to prevent errors (general practice)	O	36	1
		Practice standards	Adding to the skills of the practice	O	30	1
			Standardized questions to ask vendors	O	21, 25	2
			Model requests for proposal for contracts	O	21, 25	2
	Funding and incentive	Remunerations	Payment for having or using system	O	22, 36	3
			Payment for patient survey results or clinical quality	O	36	2
			Direct financial assistance	O	25, 38	2
		Added values				0
		Incentive programs	Financial incentives for purchase and implementation	O	21, 22, 25, 28, 35, 38	6
			Clarity of benefits	O	28	1
	Legislation, policy and governance	Legislative acts				0
		Regulations and policies	Confidentiality	O	22, 27, 28, 29	4
			Access and sharing of to medical records	O	22, 29	2
			Intellectual property regulations	O	28	1
			Self-referral prohibitions regarding sharing of technology	O	25	1
			Government regulation requiring mandatory reporting of patient information	O	28	1
		Governance bodies	Vendor certification and accreditation	O	21, 25, 38	3
			Legal liability	O	22	1
	Societal, political and economic trends	Societal trends	Competitive peer pressure in terms of more practices becoming computerized	O	28	1
			Recommendations of colleagues	O	38	1
			Public or patient views for computerization	O	26, 28, 33	3
		Political trends				0
		Economic trends				0
Micro	System	Functionality	Features available and functions computerized	O	21, 22, 25, 26, 27, 35, 39	9
			Intention to computerize functions	O	26	1
			Features desired and functions that should be computerized	O	21, 26, 29, 31, 32	10
			Features used	O	22, 26, 35, 37, 38	5
			Features for patient use	O	22	5
		Performance	Reliability of system	I	22, 34	2
			System downtime	I	27, 33	2
			Frequency of potential drug interaction alerts	I	32	1
			How good system is in alerting for significant interactions	I	32	1
			Concern system would become obsolete	O	22	1
		Security	*Security and privacy*	I	22, 25, 26, 27, 29, 33, 34, 35, 36	11
	Information	Availability	Information storage and retrieval	I	30	1
			Reliability of information	I	32	1
			*Accessibility of records and information*	I	21, 22, 24, 25, 27, 35, 36, 38	11
		Content	Value of clinical records	I	26	1
			Accuracy of records	I	21, 25, 38	3
			Drug interaction alerts providing information that is irrelevant to the patient	I	32	3
			Amount of information provided	I	32	1
			Reason for overriding alert: more faith in other sources of information	I	32	3
			Grading interaction alerts according to severity	I	32	1
	Service	Responsiveness	Training	I	24, 29, 31, 34, 38	8
			Level of support	I	28, 31, 36, 37	4
	Use	Use behavior and pattern				0
		Self-reported Use	Use of information technology for clinical management activities	O	27 (also see functionality)	1
			Overriding alerts	I	32	4
		Intention to use				0
	Satisfaction	Competency	Learning curve	O	21, 25, 27, 28, 33	6
		User satisfaction	Overall satisfaction	I	21, 22, 39	4
			Annoyance caused by drug interaction alert messages	I	32	1
			Usefulness in prescribing	I	23, 32	2
			Ease of use of system or clinical module	I	22, 23, 31, 33	5
		Ease of use	Data entry	I	25, 27, 29, 33, 38	5
			Interface and customization	I	39	1
	Net benefits	Quality: patient safety	Primary care and medical errors	I	27, 29	3
			Medication-related errors	I	22, 24, 25, 35, 36, 38	8
		Quality: appropriateness and effectiveness	Disease prevention or management	I	22, 30, 38	5
			Clinical decision making	I	22, 25	3
			Clinical functions	I	26	1
			Prescriptions	I	22, 25, 30	3
			Legibility	I	21	1
			Frequency of change in initial prescribing decision due to drug interaction alerts	I	32	1
			Awareness of information provided by drug interaction alerts	I	32	2
			Effect of computer use on patients’ satisfaction with care received	I	34	1
			*Patient–physician relationship and communication*	I	21, 22, 24, 25, 26, 27, 28, 34, 35, 36	10
			Documentation	I	27	2
			Effect on medical practice; practice style	I	39	1
		Health outcomes	*Quality of patient care or clinical outcomes*	I	21, 24, 26, 27, 28, 29, 31, 35, 36	12
		Access: ability of patients and providers to access services	Remoteness in the provision of medical care	I	30	1
			Patient or customer base and area of coverage	I	30	1
		Access: patient and caregiver participation				0
		Productivity: efficiency	Accounting and billing or charge capture	I	21, 25, 27, 30	7
			Assistance in test ordering and management	I	22, 24	3
			Documentation time	I	21, 24	3
			*Business or practice efficiency*	I	21, 27, 28, 30, 33, 34, 35, 36, 39	10
			Time for medication refills	I	38	1
			Time for patient care	I	24, 26, 30	3
			Workload	I	27, 30	4
		Productivity: care coordination	*Communication with other providers*	I	22, 24, 27, 30, 35, 36	8
			Workflow	I	21, 25, 27, 33, 37	5
		Productivity: net cost	*Costs or savings*	I	21, 25, 27, 28, 30, 35, 36	10

^a ^B = background, O = other, I = impact-specific area.

^b ^Health information system.

Our mapping of metrics to the Clinical Adoption Framework resulted in no metrics for the category patient and caregiver participation, and only 2 metrics for the ability of patients and providers to access services. Together, these make up the access category of net benefits. However, 1 paper [22] did report on available functions for patients, which could potentially be considered patient participation. However, since the survey reported on these only as available functionality within the system and not perceived impact, we classified them under other metrics for functionality. All metrics falling under functionality and competency were classified into either background or other areas.

We found appropriateness or effectiveness and efficiency to be the most-addressed areas of impact. The italicized areas in Table 4 were identified as the top impact-specific areas based on frequency of reported metrics and results in the original papers. These were security and privacy, quality of patient care or clinical outcomes, patient–physician relationship and communication, communication with other providers, accessibility of records and information, business or practice efficiency, and costs or savings.

### Selected Area Findings

**Figure 2:**
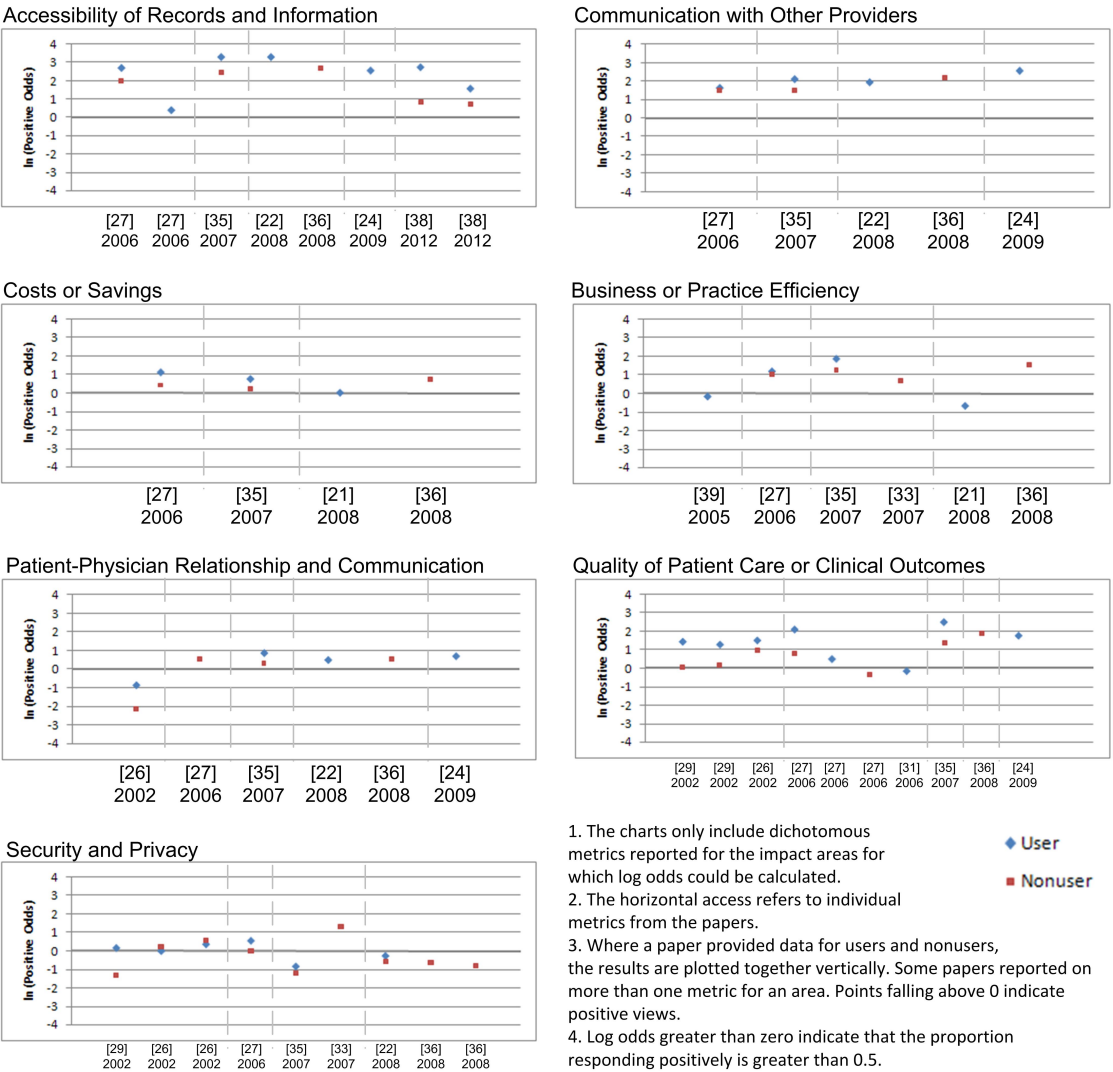
Estimated log odds for selected impact-specific areas.

We synthesized reported data for the 7 top impact-specific areas using the meta-analysis approach described above. The estimated log odds for users and nonusers are graphed in Figure 2 (see Multimedia Appendix 3 for calculations and assumptions, including proportions, standard errors, and confidence intervals).

#### Accessibility of Records and Information

Our meta-analysis showed that both users and nonusers viewed EMRs as having a positive impact on accessibility of information. For this area, 8 papers [21,22,24,25,27,35,36,38] reported on 10 different metrics. We were able to compare users with nonusers in 3 papers and found that users were more likely than nonusers to have a perceived positive view in all 3 papers. Looking only at users, we found many more positive views in 6 papers. For example, the odds of a positive view were over 3 in 3 papers [22,35,38]. The views of nonusers were also more positive in all the papers we reviewed. As well, mean scores reported in 2 papers [21,25] pointed to positive views for both users and nonusers. In both of these, improved access was the top-rated benefit.

El-Kareh et al [24] found no noted improvement in this measure from baseline to month 12 but that virtually all clinicians reported immediate improvement in this measure at study baseline.

#### Communication with Other Providers

Both users and nonusers perceived a positive effect on communication with other providers. A total of 6 papers [22,24,27,30,35,36] reported on 8 metrics for communication with other providers and health care professionals. We calculated odds ratios for 2 papers and found that, in both cases, users were likelier to hold a positive view. All the calculated odds, regardless of whether they were for users or nonusers, indicated more positive views with respect to the impact of EMRs on communication among providers. We calculated mean scores for 3 metrics reported in 1 paper [30]. All 3 scores indicated neutral to positive views. The authors in this paper also performed a series of linear regressions to determine any associated factors. They found that those who had a higher level of skill with the use of medical packages saw a greater benefit from communication with other medical organizations, and larger practices saw a greater benefit for this and communications with fellow general practitioners. El-Kareh et al [24] performed a longitudinal study over the first year following implementation and concluded that within 1 year of implementation a vast majority of clinicians felt that it improved communication among clinicians.

#### Costs or Savings

Reduction in costs or savings was generally seen as an important impact of implementation in the majority of papers we reviewed. For the meta-analysis we only included metrics that reported on impact on practice costs after implementation for net benefits. Several others assessed views on costs to implement and maintain systems, but we categorized these under return on value. For the meta-analysis we looked at 10 metrics across 7 papers [21,25,27,28,30,35,36]. We found that those with a system were more likely to have positive views of the impact on costs or savings in 2 papers [27,35] that compared users versus nonusers. Chiang et al [21] asked respondents about the effect of implementation on practice costs after 6 months and found that the systems were associated with decreased or comparable practice costs. Gans et al [25] reported several mean scores for different types of costs for users, and all indicated that the system would be a benefit in reducing costs. The one unclear result was in the study by MacGregor et al [30], where reduction of costs was seen as almost neutral, ranking as the 12th benefit.

#### Business or Practice Efficiency

In the small number of papers reviewed, improvement in business or practice efficiency was seen as a benefit of implementation. There were a total of 10 metrics reported in 9 papers [21,27,28,30, 33-36,39] addressing business or practice efficiency. In the 2 papers providing dichotomous data for nonusers and users, users were more likely to have a positive view, and both groups individually had more positive views. MacGregor et al [30] gathered categorical data on the importance of efficiency and operation as a benefit of adoption, and the results leaned toward the positive side. The authors found that those with a higher perceived level of ability with medical software packages saw higher benefits. In the study of Leung et al [28], improved efficiency was the most attractive incentive for computerizing clinical practice. In 2 papers [21,39], users indicated more negative views, but we assumed that no change in productivity was negative in one case [39] and that same, decreased, or unsure was negative in the other [21].

#### Patient–Physician Relationship and Communication

Of all papers reporting on patient–physician relationships and communication, views appeared to be generally positive, with two exceptions. For this area, 9 papers [22,24-28,34-36,] reported on 9 related metrics. The odds ratios for 2 papers produced mixed results. One paper [35] showed positive results for both users and nonusers and a higher likelihood of a positive view for users. The other paper [26] reported more negative views for users and nonusers. However, users were about 3.5 times more likely to have a less-negative view, which aligned with the authors’ conclusion that clinical users (ie, those with one or more clinical functions computerized) were less negative about potential interference with the doctor–patient encounter. The mean scores reported for users supported more positive views.

For nonusers, we calculated negative views in 2 papers. As mentioned above, in the study of Johnston et al [26], nonusers were more negative. As well, in Russell and Spooner’s study [34], nonusers felt an EMR would have a negative impact on doctor-patient interaction.

#### Quality of Patient Care or Clinical Outcomes

A total of 8 papers [24,26-29,31,35,36] reported on 11 metrics related to perceived impact on quality of care or outcomes. We found that improvement to quality of care was generally seen as a benefit of implementation in these, except for 2 papers with results indicating otherwise. In 5 papers, the odds of a perceived positive view of users versus nonusers indicated a higher likelihood of a positive view for users. The individual odds of a positive view calculated for users and nonusers were all positive as well in these papers. Looking at only user views, only 1 paper reported that users had a negative view [31].

Nonusers had more positive views for 6 out of 7 metrics according to our calculations. The exception was in the study of Kemper et al [27], where the respondents were pediatricians, and more than half of those without a system cited lack of belief that these systems improve care as a barrier to adoption. But this paper also reported that for users, a common reason for adopting was to improve quality of care. Loomis et al [29] reported that for nonusers there was a considerable lack of belief that EMRs would improve quality, but our calculations still found the odds of a positive view to be greater than 0.

One paper [28] presented a mean score indicating that respondents (users and nonusers) found higher quality of care to be the one of the most attractive incentives for computerizing their clinical practice.

#### Security and Privacy

Privacy and security appeared to be an area of mixed perceptions regarding the impact of EMRs. A total of 9 papers [22,25-27,29,33-36] addressed security and privacy through 11 metrics, which produced mixed results for positive and negative views. Out of the odds ratio estimates calculated for 6 metrics, 4 showed a higher likelihood of positive views for users over nonusers. However, this actually meant that users were more likely to have a less-negative view, because the individual odds showed more negative views in some cases. In 1 paper [35] where we found a higher likelihood of positive views for users but where each group individually had more negative views, the authors concluded that “more than 40% of responding physicians, regardless of whether they used EMRs, said that computers may have a negative effect on patient privacy.” For DesRoches et al [22], we found that those with and without an EMR system had more negative views about illegal record tampering. However, the paper interpreted this to mean that protecting physicians from personal liability for record tampering by external parties could be a major facilitator of adoption. A total of 3 papers [27,29,34] specifically reported that more users had more positive perceptions of EMRs than nonusers. Conversely, in 1 paper [26] we compared users with nonusers, where users were not more likely to have positive views. However, when looking at users and nonusers separately, we found that each group had more positive views.

When looking at only the odds of positive views for users, our calculated results for 4 out of 6 metrics were positive. For nonusers, 3 out of our 9 calculated results were positive. Mean scores were reported in 2 papers, and when these were transformed into comparable scales, they reflected positive views for both users and nonusers. For example, Russell and Spooner [34] found that neither users nor nonusers felt patient privacy was harder to ensure with an EMR but nonusers showed more concern about disadvantages regarding privacy. Gans et al [25] ranked the top barriers to implementing a system, and security and privacy were of least concern.

## Discussion

The 19 papers reviewed provided valuable insights into the state of evaluation of perceived EMR impacts through survey research methods. It is clear that evaluation from the user’s perspective is needed alongside objective measures of impact.

### Areas (Most and Least) Addressed in Survey-Based Papers

The first aim of this review was to determine which areas of EMR implementation in office practices have been addressed in survey-based research studies. The majority of background areas corresponded to the meso level, and other areas looking at aspects of implementation corresponded to the macro level. A possible explanation for the lack of metrics for individuals and groups and for roles and responsibilities is that these can be considered the basis for selecting respondents and would therefore not have specific metrics related to them. In most cases, the researchers predetermined that respondents would be physicians who may be users of the EMR. Added values, legislative acts, political trends, and economic trends are all in line with the macro level according to the Clinical Adoption Framework and certainly do affect EMR use but didn’t seem to be the main objective for surveys evaluating more localized views of implementation in the office. It may be no surprise that the expense of implementation is a major consideration for office practices, and so this was a common area addressed.

The impact-specific areas we focused on were mostly contained within the net benefits dimension at the micro level of the framework. While functionality was frequently addressed in the surveys, the questions seemed to mainly pertain to availability of features rather than impact. Future surveys may wish to ask not only what is available and desired but also how it had an impact on practice. For example, this could drill further down into the efficiency areas in that improvements in efficiency may be associated with specific functionality, such as electronic transfer of laboratory results into the record, which may eliminate paper or fax transmission and manual entry time. One might expect user satisfaction to be the most-addressed category from the Clinical Adoption Framework, as surveys do generally obtain views on satisfaction. However, to understand the specific areas of satisfaction, we teased out the aspects of user satisfaction into more specific categories so that this particular category only included overall satisfaction. For example, user satisfaction with respect to the system’s effect on their efficiency was mapped to productivity. Use behavior or pattern and intention to use may be encompassed by functions used and use status. Appropriateness or effectiveness and efficiency seemed to be well addressed through surveys, but there were areas of net benefits that would be expected to have had more metrics than were found. The reasons for the lack of addressed areas found for patient and caregiver participation aren’t apparent. Either this specific aspect of EMR use hasn’t been studied in depth or perhaps there is a degree of overlap between this category and others such as care coordination. This particular category may warrant further exploration.

### Perceived Views Among Users and Nonusers for Most-Addressed Areas

For the second contribution of this review, to compare the perceived impacts between users and nonusers, we looked at the 7 most-addressed areas of impact in the set of papers: security and privacy, quality of patient care or clinical outcomes, patient-physician relationship and communication, communication with other providers, accessibility of records and information, business or practice efficiency, and costs or savings. For these areas, the views of users were generally more positive than those of nonusers, but even when looking at the two groups separately, we found mostly positive views for most of the impact-specific areas. In reviewing computer-based patient record systems (including EMRs), Delpierre et al [40] also concluded that user satisfaction was mainly positive. We found only positive views for impact on communication with other providers and accessibility of information. The other metrics had mostly positive results with some exceptions as noted. As mentioned in the Methods section, this review focused on the survey-based studies, while another systematic review was completed on the set of analytic and descriptive studies [17]. Interestingly, that review found that only 23 of 44 studies showed overall positive impacts. While a different set of areas were examined in that paper, this suggests a gap between the perceived impacts of users and nonusers reviewed here and the actual impacts seen. For example, in terms of impact on patient-physician relationship and communication, we found that all but 1 paper indicated positive views. The systematic review [17] found that it was one area that was least improved. These findings are consistent with other reviews that have found mixed results as well. Delpierre et al [40] found that in some papers, computer-based patient record systems were perceived as a physical barrier that could have a negative impact on the patient-physician relationship. And in their review of the barriers to acceptance of EMRs, Boonstra and Broekhuis [41] found that the traditional doctor-patient relationship will be changed by EMRs, but whether it is a problem is not clear. Shachak and Reis [42] conducted a narrative literature review specifically looking at factors affecting the impact of EMRs on patient-doctor communication and concluded that EMRs have a positive influence on the information-sharing aspect of communication but negative impacts on patient centeredness, emotional and psychological communication, and establishing rapport between physicians and patients.

Privacy and security was an inconclusive area, which returned the most mixed results for users and nonusers and may therefore warrant further exploration. According to Boonstra and Broekhuis [41], many researchers agree that EMRs may have a negative effect on patients’ privacy and is a concern among both users and nonusers. The authors commented on a lack of clear security standards. To address security and privacy concerns, Loomis et al [29] suggested making systems compliant with the US Health Insurance Portability and Accountability Act and educating physicians about the security and confidentiality risks of paper records, as well as the safeguards built into EMRs.

Given that there were some noted differences in perceptions between users and nonuser with respect to most of the areas we looked at, it may be prudent to look at some of the associated background and other factors we identified in our mapping, which may account for some of the differences. Delpierre et al [40] noted in their review that system and user characteristics influenced user perceptions of the system and, in another review specifically on EMRs, the authors stated the characteristics of a practice can affect the extent of certain barriers to use [41]. A few papers in our review commented on differences seen in relation to practice size, but the results were somewhat mixed. MacGregor et al [30] saw some differences in perceived benefits related to practice size, but Gans et al [25] observed no consistent differences in benefits experienced by size of practice for practices that have electronic records. Boonstra and Broekhuis [41] discovered differences in their review and commented that further study is needed to analyze the reasons for the gap.

An interesting point noted in some of the papers we reviewed mirrors the classic chicken-and-egg puzzle. That is, did the more-positive views seen in users exist before they adopted the systems or did they develop them as a result of adoption? El-Kareh et al [24] commented that for cross-sectional studies this is not clear, and both Simon et al [35] and Russell and Spooner [34] mention it as a limitation to their studies. Russell and Spooner [34] explain that “if adopters were inclined toward EMRs to begin with then they haven’t changed attitudes because of using the EMRs but if adopters were no different from non-adopters to begin with then they have developed positive attitudes because of use.” Either way, the results support positive views among users, but this may suggest a need for more longitudinal evaluations such as that by El-Kareh et al [24].

### State of Survey-Based Research for HIS Evaluation and Quality Issues

Lastly, in assessing the state of knowledge regarding survey-based research in HIS evaluation, there appears to be a lack of clear methodological guidance. Regarding design quality, the papers varied in terms of methods used and how they were reported. The items with the highest-quality scores were reporting a profile of the sample frame, a response rate, and a profile of respondents. The item that scored most poorly across all papers was performing a reliability or validity analysis of item measurement or adopting a validated instrument. Only 1 paper specifically reported having a test–retest reliability rate for each item. The other item that generally scored low was the use of a combination of personal, telephone, and mail data collection. In most cases, survey data were collected solely through a mailed questionnaire.

In terms of constructs for evaluation, we identified an issue related to neutrality for our review. We aimed to determine whether there was an overall positive or negative perceived impact with respect to each selected area but found that, in many cases, the individual survey items seemed to lean in one direction or the other—for example, a survey item asking respondents whether security is a barrier versus an item asking whether the respondent sees a benefit with regard to security. Both items address the construct of security, but the responses elicited by each may be affected by how they are posed. Therefore, it is possible that the constructs for evaluation used in each study may have had an impact on the negative and positive responses collected, which in turn affects how the results are reconciled across papers. In designing surveys, Trochim [43] asks the reader to consider what assumptions the question makes and whether the wording is loaded or slanted. In this case, where respondents are being asked for their opinion regarding an evaluation item, it may be better to ask the question in a more neutral manner, such as “What do you think about the system in regard to security?” Respondents can then rank their opinion on a Likert scale ranging from negative to positive. Or, for dichotomous data, the question can be posed as “What effect do you think the system would have on security?” The choices can be positive or negative so that the overall percentage of respondents indicating each type can be calculated and reported. An example of a paper in our review that did present neutral results well was that by Simon et al [35]. Table 2 in that paper provides percentages of respondents indicating a positive, negative, or no perceived effect of computers according to 8 dimensions. The results are presented for electronic record adopters (broken down further by high- and low-use adopters) and nonadopters. *P *values are included in the table to show significant differences between adopters and nonadopters. Although the data are presented as percentages, sample sizes are provided for each group so that proportions can be estimated.

### Limitations

We experienced several challenges common to meta-analyses of survey-based research. At the paper selection stage, a major challenge was determining whether the paper discussed EMR use, which relied on descriptions of functionality provided in the papers. This review is based on a small set of 19 papers, and we had to further narrow down the set of papers for the meta-analysis based on the type of data reported. The biggest limitation in our review is related to the heterogeneity of all the papers included. Both Rao et al [14] and Jamal et al [15] describe this challenge as well in their respective reviews. In mapping the metrics and areas, we based categorizations on what was reported, which was sometimes nothing more than a simple term. Several assumptions were made for calculations as noted, and perhaps a key consideration to note is that the estimates produced don’t necessarily reflect statistically significant results. They are best estimates based on the reported data, intended to help produce indications rather than absolute measures. As mentioned above regarding constructs for evaluation, we had to make assumptions about one-sided (negative or positive) results reported in order to be able to compare and synthesize them. For example, a nonnegative result was assumed to be positive. Sometimes there were several metrics for an area, but they all came from the same paper. We relied solely on the information reported in the papers and didn’t seek out all survey tools to analyze at the individual question level because one of the goals was to determine what the focus of prior survey research has been through what has been reported. Finally, there is the possibility of publication bias in our source papers, which may have contributed to the number of positive results seen. Despite these many challenges, we were still able to devise and follow a systematic, rigorous approach to synthesize the data from our set of survey-based papers to produce some interesting results.

### Conclusion

Although based on a small set of papers and estimated calculations, the results of this review are promising in terms of clinicians’ views for adoption of systems and suggest that clinicians are beginning to see benefits in certain areas. However, there are additional factors (eg, organizational and system) that influence perceptions, so it is important to consider a wide range of contextual factors when adopting an EMR. Our mapping identified areas corresponding to categories of the Clinical Adoption Framework that have been addressed most and other areas that haven’t been looked at yet for evaluation through surveys. Although practices with electronic record systems already in use may have more positive views of impact, our review found that those without the systems still generally have a perceived positive impact with respect to some key areas, with the exception of mixed views toward privacy and security. The findings of this review have the potential to highlight areas of concern or benefit for adoption and should be considered in future implementations and evaluations. One hope is that nonusers can look to the areas where users saw more positive perceptions as areas where they can expect to see potential benefits for adoption. As well, associations between the most-addressed areas and least-addressed areas may help practices determine where they can focus effort during implementation planning, taking into account some of the key background and other areas we’ve identified.

Survey-based research studies are a valuable way to collect users’ views for HIS evaluation. They offer data and findings that can make a significant contribution to the field. However, careful effort should be made to ensure methodological rigor and consider potential future syntheses. Our review demonstrated an approach for reconciling results presented in different ways across heterogeneous survey-based studies, which is a recognized challenge. In terms of design quality, researchers should ensure that important survey-based research study design elements are present and clearly reported using a guide such as the survey methodological attributes. As well, the constructs for evaluation should draw from an established framework or tool when possible and be expressed in a neutral manner to elicit peoples’ views.

## Multimedia Appendix 1

Paper selection flow diagram.

## Multimedia Appendix 2

The Clinical Adoption Framework.

## Multimedia Appendix 3

Estimated odds and mean calculations for selected impact-specific areas.

## Abbreviations

EMR: electronic medical record
HIS: health information system
