# Supplementary figures and images for: Perceived Impact of Electronic Medical Records in Physician Office Practices: A Review of Survey-Based Research
Source: Interact J Med Res. 2012 Jul 28;1(2):e3. doi: 10.2196/ijmr.2113 (PMC3626136; doi:10.2196/ijmr.2113)

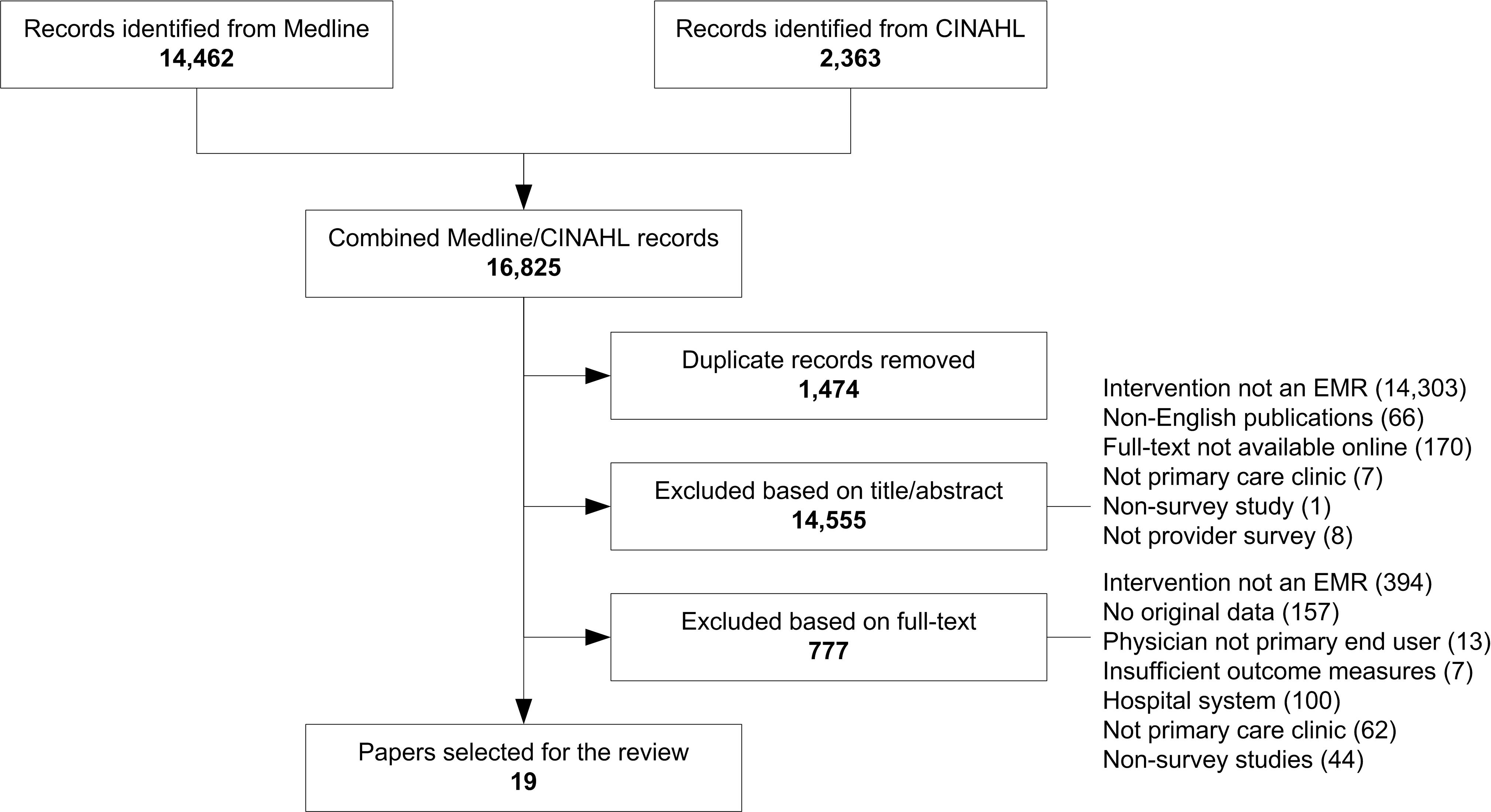

Supplement: Supplementary file 1 [file ijmr_v1i4e3_app1.jpg]

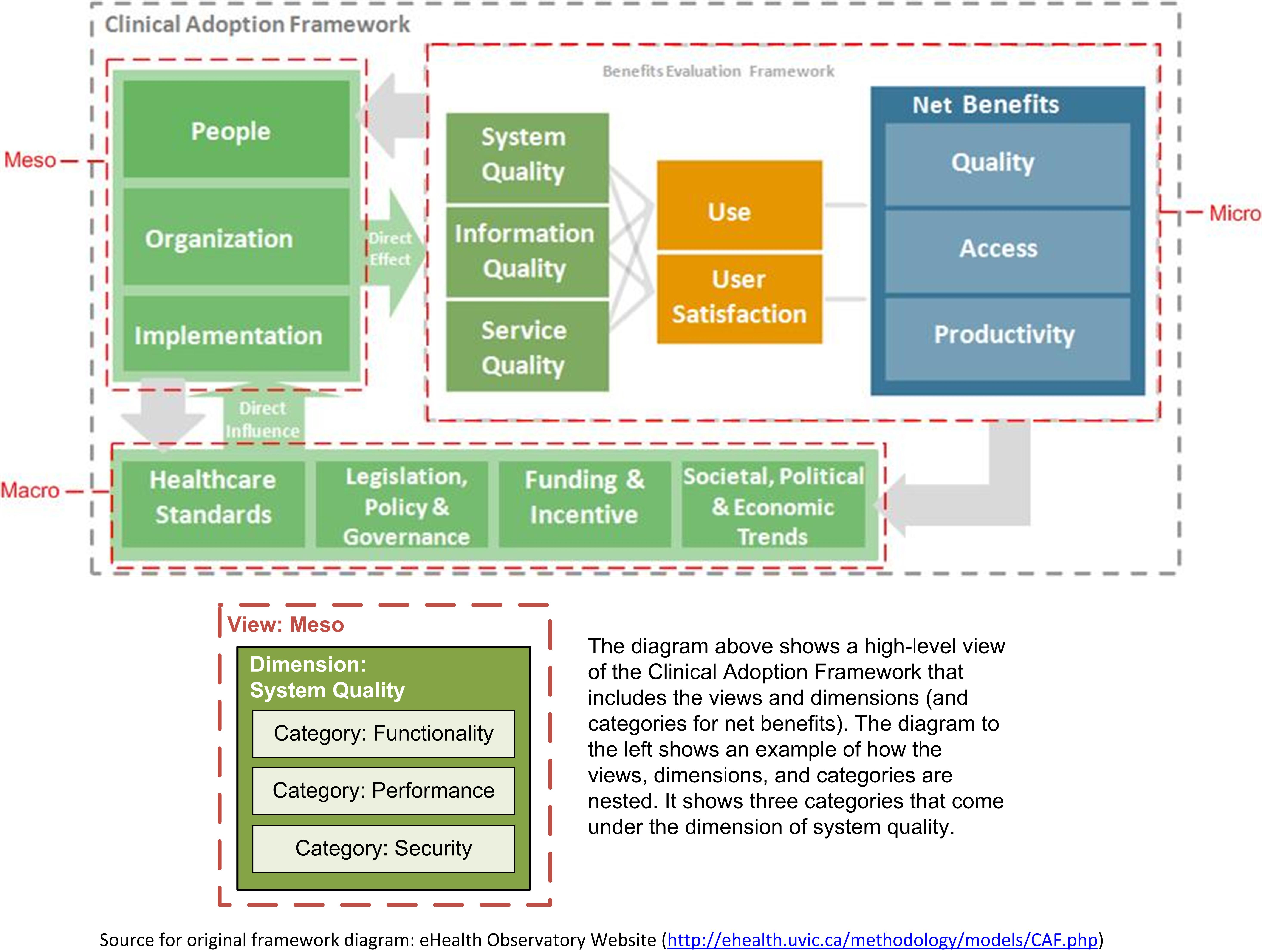

Supplement: Supplementary file 2 [file ijmr_v1i4e3_app2.jpg]
